# Natural compounds as potential countermeasures for spaceflight-associated neuro-ocular syndrome

**DOI:** 10.3389/fopht.2026.1801651

**Published:** 2026-10-09

**Authors:** R. Mikal Lange, Tasneem P. Sharma

**Affiliations:** 1 Department of Ophthalmology, Eugene and Marilyn Glick Eye Institute, Indiana University School of Medicine, Indianapolis, IN, United States; 2 Department of Biochemistry, Molecular Biology and Pharmacology, Indiana University School of Medicine, Indianapolis, IN, United States

**Keywords:** coenzyme Q10, space biology, spaceflight-associated neuro-ocular syndrome, stress, supplement, oxidative stress, inflammation

## Abstract

Spaceflight-Associated Neuro-Ocular Syndrome (SANS) is a complex and multifactorial condition most commonly observed following long-duration spaceflight. Clinical findings of SANS include optic disc edema, retinal nerve fiber layer swelling, globe flattening, chorioretinal folds, and hyperopic refractive shifts. As human space exploration extends toward prolonged missions such as Artemis and Mars expeditions, SANS represents a significant risk to astronaut health and mission success. Although headward fluid shifts, elevated intracranial pressure, venous outflow congestion, and lymphatic dysfunction have been implicated in SANS pathophysiology, no single unifying mechanism or universally effective countermeasure has been identified. Emerging evidence suggests that oxidative stress from galactic cosmic radiation, endothelial dysfunction, inflammation, mitochondrial injury, altered angiogenesis, and disruptions in one-carbon metabolism contribute meaningfully to SANS development. This review summarizes the clinical manifestations and proposed mechanisms underlying SANS and examines naturally occurring compounds with high therapeutic indices that target these pathways. Specifically, we review bilberry anthocyanins with French maritime pine bark extract, Ginkgo biloba extract, saffron/crocin, curcumin, coenzyme Q10, resveratrol, vitamin E, and B vitamins as potential supplemental approaches, highlighting their mechanisms of action and therapeutic efficacy when targeting terrestrial ocular diseases. While direct studies in spaceflight environments remain limited, these compounds demonstrate antioxidant, anti-inflammatory, neuroprotective, vasoregulatory, and metabolic effects relevant to SANS. Collectively, they represent promising adjunctive candidates within a multimodal preventative strategy aimed at mitigating SANS risk during long-duration spaceflight.

## Introduction

### Clinical findings of Spaceflight-Associated Neuro-Ocular Syndrome

Spaceflight-Associated Neuro-Ocular Syndrome (SANS) is a unique collection of neuro-ocular clinical findings typically associated with long-duration spaceflights (LDSF), which is defined as spaceflight that lasts 30 days or longer (1). With clinical signs documented for over a decade, SANS is of paramount concern in space medicine, especially for long term operations such as the Artemis and Mars missions. These SANS findings include optic disc edema (ODE), retinal nerve fiber layer (RNFL) swelling, globe flattening (GF), chorioretinal folds (CF), and hyperopic refractive error shifts ranging from 0.50 to 1.75 diopters (1). Fortunately, most of these findings are reversible after short duration flights; however, several studies have reported permanent clinical findings of SANS after returning to Earth, including refractive error shifts, CF, and GF (2–4). In Mader’s 2011 study that first described SANS, the primary complaint among astronauts was a progressive worsening of near visual acuity as quickly as three weeks into the mission. (1). It has been reported that 48% of astronauts note hyperopic shifts after LDSF, compared to just 23% after short duration spaceflight (SDSF), illuminating a correlation between this symptom and spaceflight duration (1). There have been a few reports of decreased distance visual acuity and visual scotoma findings as well (1, 4).

### Pathophysiology and potential mechanisms of SANS

SANS has been identified by the National Aeronautics and Space Administration (NASA) as a high-priority risk for future space exploration, and understanding its pathophysiology and mitigating its effects is essential for the success of future missions (5). A clear mechanism for the development of SANS has yet to be proven. Theories regarding the pathogenesis of SANS are often not mutually exclusive, making the identification of a unifying treatment for SANS difficult. Several studies have focused on the headward fluid shift and subsequent elevated intracranial pressure (ICP) in the microgravity environment (6–9). Similarly, several studies have focused on venous outflow congestion and lymphatic outflow dysfunction as possible etiologies (10–12). Additional etiologies that may be targeted by naturally occurring compounds include one-carbon metabolic pathway abnormalities, sleep deprivation, carbon dioxide exposure, and oxidative stress from exposure to galactic cosmic radiation (GCR) (12).

Most countermeasures for SANS focus on addressing the elevated ICP and venous outflow congestion associated with the microgravity environment; however, relatively fewer studies have examined various naturally occurring compounds with high therapeutic indices as potential countermeasures to target these detrimental effects. As our understanding of SANS grows, we become increasingly aware that metabolic, oxidative, inflammatory, and microvascular pathways play important contributory roles to SANS pathophysiology. (12). We hypothesize that the mitigation of SANS will likely require a multifactorial approach that addresses each of these pathways without causing significant side effects. Therefore, in this review we will discuss the clinical signs of SANS, present various natural compounds that have been studied in other ocular diseases, their mechanisms of action, and their potential therapeutic efficacy in treating SANS.

### Oxidative stress in SANS

Exposure to microgravity and GCR induces a robust oxidative stress response across multiple organ systems, driven largely by the increased production of reactive oxygen species (ROS) and impaired antioxidant defenses (13–15). Terrestrial microgravity analogues and spaceflight studies demonstrate mitochondrial dysfunction, altered redox signaling, and accumulation of oxidative damage to DNA, proteins, and lipids in neural, cardiovascular, musculoskeletal, and immune tissues (15). GCR, particularly high-energy charged particles, further exacerbates oxidative injury through direct ionization events and secondary ROS generation, producing sustained oxidative stress even at relatively low radiation doses (16). These combined stressors are considered central contributors to oxidative stress that causes tissue degeneration during LDSF (13, 16, 17).

Outside of oxidative stress, microgravity and GCR also provoke vascular dysfunction and maladaptive inflammatory responses, which may play a role in the development of SANS. Microgravity alters endothelial cell morphology, cytoskeletal organization, and nitric oxide signaling, leading to impaired vasoregulation, increased vascular permeability, and endothelial dysfunction (18). Simultaneously, spaceflight conditions dysregulate immune signaling, promoting chronic low-grade inflammation characterized by altered cytokine profiles and immune cell activation (19). These inflammatory pathways may amplify oxidative stress and vascular injury, potentially worsening tissue damage (20). Collectively, these mechanistic insights provide a strong biological rationale for targeting oxidative and inflammatory pathways with antioxidant and anti-inflammatory countermeasures as potential strategies to mitigate SANS.

### Mechanical and fluid dynamic mechanisms in SANS

On Earth, hydrostatic pressure gradients shift approximately 1–2 L of fluid toward the lower extremities in the upright posture, maintaining relatively low ICP. In microgravity, the loss of these gradients results in a sustained cephalad fluid redistribution (9). While early hypotheses proposed that this fluid shift would produce pathology analogous to terrestrial intracranial hypertension, current evidence from lumbar puncture data in astronauts and ground-based analogues suggest that ICP in microgravity is only mildly elevated but remains elevated chronically due to an inability to assume an upright posture (6). This persistent mild elevation reduces the translaminar pressure gradient, which is the pressure difference across the lamina cribrosa between IOP and ICP, which may contribute to the posterior globe flattening, ODE, and remodeling of the lamina cribrosa seen in SANS (9).

Concurrently, microgravity impairs cranial venous outflow. Stagnant retrograde internal jugular venous flow has been documented in astronauts during LDSF, with one case of in-flight jugular vein thrombosis reported (10, 21). Astronauts who developed SANS demonstrated greater increases in dural venous sinus volumes compared to those who did not, suggesting that individuals with increased venous sinus compliance may be at risk of developing SANS (22). A recent analysis has also proposed that microgravity-induced venous overload of the choroid may represent a distinct mechanism contributing to choroidal thickening and choroidal folds that are not fully explained by elevated ICP alone (23).

Emerging evidence also implicates glymphatic dysfunction in SANS. MRI studies have demonstrated that perivascular space volumes in both white matter and basal ganglia increase from preflight to postflight, and the degree of perivascular space enlargement has been associated with SANS findings (24). Impaired cerebral venous outflow and compromised CSF resorption may obstruct glymphatic perivenous drainage while increasing periarterial CSF inflow, leading to fluid accumulation in perivascular spaces (24). The ocular glymphatic system may be similarly affected, potentially contributing to SANS pathology (25). One proposed hypothesis is that glymphatic impairment may represent a common downstream pathway through which multiple SANS risk factors, including fluid shifts, hypercapnia, and genetic predisposition, converge (25).

Furthermore, the structural changes observed in SANS raise important questions about the biomechanical forces acting on the globe and optic nerve head. ODE is detected in approximately 70% of crew members during LDSF and develops as early as flight day 30 and progresses throughout the mission, with maximal changes occurring even beyond a six-month mission (26, 27). Crowded optic nerve head morphology, such as a small preflight optic cup volume, has been identified as a potential risk factor for developing more severe ODE during LDSF, suggesting that individual anatomic variation may play a role in susceptibility to SANS (27).

Notably, the pattern of ocular deformations in SANS differs significantly from that seen in idiopathic intracranial hypertension (IIH). Changes include the Bruch’s membrane deformations being small and bidirectional in SANS, whereas they are large and mostly anterior in IIH, further indicating the etiology of SANS is likely more than just elevated ICP alone (28). An additional study by Reilly et al. proposed that anteriorly directed force from orbital fat swelling in microgravity may produce a larger mechanical effect on the globe than ICP elevation, potentially serving as a unifying mechanism for globe flattening, decreasing axial length, and loss of peripapillary choroidal tension (29). While many of the structural changes that occur with SANS resolve after returning to Earth, some changes persist well after astronauts have returned. Some astronauts have experienced persistent axial length shortening and hyperopic refractive shifts that have been documented up to one-year postflight, and summated brain and CSF volumetric expansion may not fully recover within this timeframe (26, 30).

These mechanical, fluid dynamic, and structural mechanisms are not mutually exclusive from the oxidative, inflammatory, and metabolic pathways implicated in SANS pathogenesis. More probably, they likely interact, such as venous congestion exacerbating oxidative stress in retinal tissues, and inflammatory mediators worsening vascular permeability in the setting of altered fluid dynamics, resulting in the multifactorial pathophysiology of SANS (12). For a summary of SANS findings, their diagnostic modalities, frequency in SANS, and severity, see **Table 1**.

**Table 1:** SANS findings incidence and diagnostics.

SANS finding	Primary diagnostic modality	Reported frequency (LDSF)	Severity/quantitative metrics	References
Optic disc edema (ODE)	OCT (peripapillary total retinal thickness [TRT] within 250 µm of Bruch membrane opening; minimum rim width [MRW]); ophthalmoscopy/fundoscopy for clinical grading	~16–19% by ophthalmoscopy (clinically relevant ODE); higher by sensitive OCT (subclinical thickening)	ODE threshold ΔTRT ±19.4 µm; global TRT increased 8.5% (362 to 393 µm); MRW/TRT rise as early as FD10, maximal near FD150	(26, 27, 31)
Chorioretinal folds (CF)	OCT with Bruch membrane surface-roughness analysis; fundoscopy	Subset of crew; when folds occur, choroidal folds present in all affected eyes	Surface roughness change threshold 2.8 µm; predominantly nasal, superior, inferior peripapillary sectors, sparing temporal	(32, 33)
Globe flattening (GF)	MRI (orbital); OCT; ocular ultrasound	Subset of crew (5/7 in original cohort)	Posterior globe flattening; axial length decrease −0.09 mm postflight	(1, 34, 35)
Hyperopic refractive error shift	Cycloplegic/manifest refraction; visual acuity testing	~48% after LDSF vs ~23% after SDSF; 48.2% of eyes hyperopic shift	+0.50 to +1.75 D spherical equivalent; mean +0.12 D from AL −0.09 mm	(1, 34, 35)

Clinical findings of SANS, the primary diagnostic modalities used for detection and monitoring, reported frequency during LDSF, and associated severity or quantitative metrics. ODE is the most common finding and can be detected by OCT earlier and more sensitively than by ophthalmoscopy, accounting for the discrepancy between clinically graded and OCT-based frequencies. Quantitative thresholds (e.g., change in peripapillary total retinal thickness, Bruch membrane surface roughness) reflect values reported in the cited astronaut cohorts. Frequency estimates should be evaluated cautiously given small sample sizes and variation in imaging protocols and diagnostic criteria across studies. Abbreviations: SANS, spaceflight-associated neuro-ocular syndrome; OCT, optical coherence tomography; TRT, total retinal thickness; MRW, minimum rim width; MRI, magnetic resonance imaging; ODE, optic disc edema; CF, chorioretinal folds; GF, globe flattening; AL, axial length; D, diopters; FD, flight day; LDSF, long-duration spaceflight; SDSF, short-duration spaceflight.

### Terrestrial ocular diseases as a starting point for SANS

Although SANS is a distinct clinical entity, the compounds discussed in this review have been studied predominantly in terrestrial ocular diseases, primarily glaucoma, age-related macular degeneration (AMD), and diabetic retinopathy (DR). These diseases are relevant to SANS not because of a shared common etiology, but because they converge on the same downstream pathophysiological pathways that are implicated in SANS: oxidative stress, endothelial and microvascular dysfunction, inflammation, and in the case of B-vitamins, disruption of one-carbon metabolic pathways (12). Each of these pathways can be linked to specific SANS findings. Oxidative and mitochondrial injury to retinal ganglion cells (RGCs) is well characterized in glaucoma models which parallels the retinal nerve fiber layer swelling and ODE observed in SANS. This provides a rationale for investigating compounds with established neuroprotective effects like CoQ10, curcumin, saffron/crocin, and vitamin E. The endothelial dysfunction and impaired ocular perfusion seen in DR and studied with Mirtogenol and Ginkgo biloba extract correspond to the venous outflow congestion, choroidal engorgement, and vascular changes described during LDSF (23, 36).

These parallels should not be interpreted as shared diseases, rather they should be considered as similar mechanisms. Several important discontinuities should be acknowledged. Unlike glaucoma, SANS is not primarily driven by elevated IOP, as IOP is only transiently and mildly elevated in spaceflight (37). Thus, the IOP-lowering effects reported for some of these compounds is of limited relevance, and only their neuroprotective and vasoregulatory mechanisms are likely to be beneficial. Unlike AMD, which is a chronic degenerative disease of the retina, SANS is a largely reversible syndrome driven by a confluence of fluid shifts, altered pressure gradients, and GCR exposure. Most notably, whereas wet AMD and DR are characterized by pathological neovascularization, SANS may instead involve retinal vascular rarefaction, which would make anti-angiogenic properties counterproductive (38). Thus, it should be noted that not all therapeutic benefits seen in terrestrial ocular diseases may be transferable to SANS and are only extrapolations requiring validation in spaceflight or analogue models.

## Subsections

### Natural compounds with potential in treating SANS

In the space environment, limited medical resources and the inability to manage the adverse effects of medical interventions make it paramount that any interventions cause minimal to no adverse effects (39, 40). Astronauts face unique spaceflight hazards, including microgravity and GCR, that cause altered pharmacokinetics, and the limited access to medical care that can make even mild side effects more consequential and render adverse events much more difficult to manage during LDSF (17, 40, 41). Thus, naturally occurring compounds with high therapeutic indices are attractive candidates, as they may confer benefit with minimal risk of harm. An important distinction should be drawn, however, between two potential roles for these agents. Primarily, they are best positioned as prophylactic countermeasures administered before and during spaceflight to attenuate the oxidative, inflammatory, and microvascular injury that drives SANS and therefore could potentially reduce its incidence and severity in susceptible crew members. The terrestrial literature reviewed below primarily supports that these compounds could slow or reduce the onset of disease rather than reverse established pathology; however, some of these compounds (saffron/crocin, Mirtogenol, and resveratrol) additionally carry human evidence of improving established terrestrial ocular disease, suggesting a possible therapeutic role once SANS findings have developed, although any disease-modifying effects transferring to SANS is purely speculative. Notably, because these compounds exert effects across multiple organ systems, they may simultaneously address the underlying mechanisms of SANS in addition to other spaceflight-induced pathologies.

### Bilberry anthocyanins and French maritime pine bark (Mirtogenol^®^)

Mirtogenol is a combined therapy of bilberry anthocyanins, acquired from bilberry extract, and French maritime pine bark extract. Mirtogenol’s mechanism of action is multifaceted, as it has antioxidant activity through the scavenging of ROS to reduce lipid peroxidation, enhancing endothelial function via increased nitric oxide (NO) production, stabilizing leaky capillaries in the retina by binding proteins and mucopolysaccharides in their walls, and improving microvascular blood flow via relaxation of blood vessels (42, 43). Bilberry anthocyanins and pine bark procyanidins are thought to upregulate enzymes like superoxide dismutase and glutathione peroxidase while simultaneously downregulating pro-oxidant enzymes like NADPH oxidase. The increased NO production activates intracellular guanylyl cyclase, increasing intracellular cGMP leading to vasorelaxation. The combination of Mirtogenol’s antioxidant and vasodilatory properties likely results in the improvement in vascular perfusion seen in the treatment of intraocular hypertension (44).

Mirtogenol has been studied primarily in glaucoma and intraocular hypertension, with some studies focusing on DR. One prospective, controlled trial using 38 human subjects found that Mirtogenol was able to reduce elevated IOP from about 25.2 mmHg to around 22.0 mmHg over 2–3 months, with significantly improved ocular blood flow in the central retinal, ophthalmic, and posterior ciliary arteries compared to baseline and control (44). In a Wistar glaucoma model, Mirtogenol was found to reduce IOP as well as significantly reduce retinal ganglion cell (RGC) apoptosis (42). In DR, pine bark procyanidins were shown to reduce retinal edema and improve vascular flow early in disease, creating a favorable prognosis for patients’ vision (45).

It is important to note that while a transient increase in IOP occurs upon entry into microgravity, chronic pathological IOP elevation has not been observed during LDSF. The largest in-flight study to date found that IOP during LDSF was elevated by only 1.3 mmHg compared to preflight values, which is not likely to be a clinically significant change (37). Earlier short-duration Space Shuttle data showed larger acute IOP increases, with one study finding that normotensive IOP astronauts all experienced increased IOP in-flight compared to pre-flight, with the average increase being 36% of pre-flight IOP (n = 3) (46). This may reflect a transient response to entering microgravity, followed by normalization via a compensatory decrease in aqueous humor volumes (47). Therefore, the rationale for Mirtogenol in SANS does not rest on its ability to lower IOP, but more so it rests on its antioxidant, anti-inflammatory, and vasoprotective properties.

Current clinical evidence indicates that Mirtogenol has a favorable safety and tolerability profile, particularly in patients that experience elevated IOP. One prospective controlled clinical trial of asymptomatic patients with elevated IOP found no observed adverse effects in the group treated with Mirtogenol over six months (44). Individually, French maritime pine bark extract has demonstrated a high safety margin in multiple human studies, but some studies report mild gastrointestinal (GI) discomfort in GI sensitive patients treated with pycnogenol (48). The manufacturers of pycnogenol report that GI discomfort can be prevented by taking pycnogenol with meals. In another study that examined bilberry extract alone, the investigators found no increased risk of adverse events compared to placebo (49). Collectively, this data supports Mirtogenol as a well-tolerated supplement. With Mirtogenol’s strong safety profile and its ability to improve vascular function and scavenge ROS in terrestrial ocular pathologies, it is worth investigating in space analogue models as a possible therapeutic agent and countermeasure for SANS.

### Ginkgo biloba extract

The most common Ginkgo biloba extract (GBE), EGb 761, contains flavonoids and terpenoids that exert antioxidant, anti-inflammatory, and vasoactive effects. The flavonoids scavenge ROS and reactive nitrogen species (RNS), while terpenoids decrease free radical release and upregulate reducing enzymes such as superoxide dismutase, glutathione peroxidase, catalase, and heme-oxygenase 1 to process free radical molecules (50). GBE has also been shown to promote erythrocyte deformability, increase NO production, and decrease fibrinogen levels; thus, GBE improves blood viscosity, blood flow, and inhibits microthrombus formation, which improves ocular perfusion (50–52). Additionally, GBE suppresses inflammation by regulating the protein-1 signaling pathway to decrease TNF-α as well as downregulating NF-κB, which lowers levels of Prostaglandin E2 (PGE2) (53, 54). The combined effects of flavonoids and terpenoids in GBE help protect neural tissue, including RGCs.

GBE has been studied in several ocular diseases, particularly both normotensive glaucoma (NTG) and hypertensive glaucoma. In patients with NTG, it is possible that GBE helps to preserve visual fields (VFs) over time. One study tested a long-term effect of GBE on VFs showed significant improvement in VFs over four years, as evaluated by a Humphrey Visual Field Analyzer. They found that both the regression coefficient and mean total deviation improved when measured before and after GBE treatment, with no significant change in IOP across all participants, pointing toward increased ocular blood flow or cognitive function as the source of VF improvement (50, 55). This is contrasted by a study by Guo et. al., which looked at 28 patients in a prospective, placebo-controlled crossover study who received GBE or a placebo over a four-week period (56). This study did not detect a significant difference in VFs. Despite the controversy surrounding VF preservation, it is well established that GBE decreases RGC loss in glaucoma models (50, 57, 58).

Other studies on GBE in glaucoma patients have demonstrated increased ophthalmic artery end-diastolic velocity as well as increased blood flow velocity in the retrobulbar vasculature, superior, and inferior capillaries when compared to a placebo group (59, 60). Additionally, rodent studies have found that GBE administration increased blood flow to the choroid via enlarged vessel caliber and elevated flow rate (61). If ocular blood flow can be improved, it is possible this may aid with the venous outflow congestion that is identified in SANS (36).

Of note, many studies monitoring the safety of GBE have found minimal adverse effects within a prescribed dosage range (62–64). Certain self-reported adverse events include headache, dizziness, constipation, palpitations, and allergic skin reactions; however, systematic reviews of the Cochrane database, PubMed/Medline, and EMBASE databases report no significant difference in adverse events between placebo and 80–600 mg of GBE (50). A systematic review by Bent et al. found a possible causal association between GBE and increased bleeding risk (65), but assessment of 29 coagulation parameters was unable to show evidence of changes in blood coagulation or platelet aggregation in 50 healthy, male volunteers who were treated with GBE (66). For a clear summary of GBE’s effects across multiple parameters and how these effects might impact SANS, see **Table 2**. Collectively, through its antioxidant and anti-inflammatory properties, improvement of ocular and retrobulbar blood flow, and potential mitigation of venous congestion, GBE warrants further investigation as a potential countermeasure for SANS, though no spaceflight or analog studies have been conducted.

**Table 2:** Dosage and effects of GBE.

Effect/outcome	Direction	Model/condition	Dose	Reference
Visual field preservation	Positive	Human, NTG, retrospective	80 mg BID (160 mg/day)	(55)
Visual field/contrast sensitivity	Null	Human, NTG, RCT crossover	40 mg TID (120 mg/day)	(56)
Ocular blood flow velocity	Positive	Human, vs placebo	40 mg TID (120 mg/day)	(59)
Retrobulbar/capillary blood flow	Positive	Human OAG, RCT crossover	Multi-ingredient supplement; discrete GBE dose not reported	(60)
Hemorrheologic improvement	Positive	Human T2DM with retinopathy	EGb 761 oral; mg/day not stated in record	(51)
Coagulation/platelet aggregation	Null	Healthy male volunteers	120 mg BID (240 mg/day)	(66)
Bleeding risk	Negative/cautionary	Systematic review	Cases at 120–160 mg/day	(65)
Adverse events overall	Null (no difference vs placebo)	Systematic review	80–600 mg	(50)

Reported effects of Ginkgo biloba extract (GBE) on ocular and hemostatic outcomes, organized by outcome, direction of effect, study model or condition, and dose. The “Direction” column indicates whether the intervention produced a beneficial (positive), neutral (null), or adverse/cautionary (negative) effect relative to control or baseline for the outcome studied. Studies span human clinical trials, healthy-volunteer studies, and systematic reviews. Doses are reported as administered in each source. The heterogeneity of study designs, populations, formulations (e.g., standardized EGb 761), and dosing makes direct cross-study comparison difficult, and results should be interpreted as a qualitative summary of the available evidence rather than pooled estimates. Abbreviations: GBE, Ginkgo biloba extract; NTG, normal-tension glaucoma; OAG, open-angle glaucoma; RCT, randomized controlled trial; T2DM, type 2 diabetes mellitus; BID, twice daily; TID, three times daily.

### Saffron/crocin

Saffron is a spice that is comprised of dried stigmas from the *Crocus sativus* L. flower, which is a stemless herb that belongs to the Iridaceae family. Its pharmacologically active constituents include bitter principles (i.e. picrocrocin), volatile agents (i.e. safranal), and dye materials (i.e. crocetin) (67). Saffron has been used in herbal medicine to treat a variety of disorders, including depression, anxiety, cardiovascular disease, diabetes, as well as various ocular pathologies (67). Its diverse therapeutic potential is derived from multiple pharmacological mechanisms.

Saffron can activate the NRF2 pathway, a key transcription factor that controls the expression of antioxidant genes. By activating this pathway, saffron prompts cells to produce more antioxidant enzymes, such as glutathione peroxidase, SOD, and catalase (68). Similarly, crocin and crocetin suppress the pro-inflammatory NF-κB pathway via inhibiting the Dermatophagoides farinae extract (DfE)-induced phosphorylation and degradation of IκBα, a signaling molecule that leads to the activation of NF-κB (69). Furthermore, crocin and crocetin were shown to enhance oxygen diffusion and improve ocular blood flow in rat and rabbit studies (70). Finally, saffron and its constituents have been shown to enhance insulin sensitivity and prevent pancreatic beta cell failure, which improves hyperglycemia (71, 72).

Several studies have examined the use of saffron in ocular pathologies (71, 73–76). In studies on patients with AMD, all doses of saffron supplementation were shown to preserve both objective and subjective visual acuity as determined by ERG and Snellen chart data, respectively (73, 74, 76). In one study focused on primary open-angle glaucoma, saffron was found to significantly decrease IOP after just three weeks when used as an adjunct to timolol and dorzolamide (75). To date, there has not been an investigation of the effects of saffron alone on IOP. Finally, saffron has been shown to be beneficial in diabetic maculopathy. One study by Sepahi et al. demonstrated that 15 mg daily doses of saffron improve central macular thickness, best corrected visual acuity, hyperglycemia, and HbA1c after three months of treatment (71).

Saffron has a relatively high therapeutic index. While there are relatively few studies that examine the safety profile of crocin, the ones that have been performed have shown that saffron doses < 1.5 grams/day are not associated with any increase in adverse events compared to placebo (67). In studies that examined saffron as a therapeutic for ocular diseases, the doses tend to range from 15–50 mg/day (67). Through activation of endogenous antioxidant defenses, suppression of inflammatory signaling, improvement of ocular blood flow, and modulation of metabolic stressors, saffron and its bioactive constituents may have therapeutic potential to mitigate oxidative, vascular, and inflammatory mechanisms implicated in the development of SANS with minimal risk of toxicity and should be investigated in spaceflight analogue models.

### Curcumin

Curcumin (diferuloylmethane) is a polyphenol derived from *Curcuma longa* L. that has been used for thousands of years in traditional Chinese medicine and Ayurvedic medicine to treat liver diseases, diabetes, atherosclerosis, infectious diseases, and cancer (77). Its diverse efficacy in treating a variety of pathologies is due to curcumin possessing antioxidant, anti-inflammatory, antimutagenic, antimicrobial and anticancer activities (77).

Curcumin serves as an antioxidant via multiple mechanisms. It serves as a scavenger of ROS and RNS, as well as enhances the activity of glutathione (78–80). Curcumin also helps to eliminate ROS by increasing the synthesis of ROS-neutralizing enzymes like catalase and SOD (81). Furthermore, curcumin blocks ROS generation by enzymes like lipoxygenase/cyclooxygenase and xanthine hydrogenase/oxidase (81). Its anti-inflammatory properties are mediated by blocking the activation of the NF-κB pathway (82). It also induces the expression of heme oxygenase (HO-1), which is essential in the cellular response to oxidative stress and angiogenesis (83). Curcumin has been shown to inhibit angiogenesis through multiple pathways; however, while angiogenesis must be limited in wet AMD, DR, and other pathologies, it is likely to be more harmful in SANS. One study found that 11/16 retinas from astronauts experiencing SANS returned with decreased fractal density and vessel length in small retinal arteries, suggesting that SANS may involve vascular rarefaction rather than neovascularization (38). Whether curcumin’s anti-angiogenic properties could exacerbate this microvascular loss remains unknown and warrants investigation. Regardless, curcumin’s safety profile and potential in treating ocular diseases makes it worth investigating as a potential preventative therapy for SANS, though its anti-angiogenic effects should be carefully evaluated in spaceflight analogue models.

Curcumin has been studied as a potential therapeutic for ocular diseases within *in vivo* rat models and cellular models. In a study on rats with chronic high IOP, the investigators found that curcumin significantly increased cell viability of BV-2 microglia (84). In a model of glaucoma that utilized increased IOP followed by reperfusion (I/R), it was found that dietary curcumin was neuroprotective by decreasing mitofusin 2 (a mitochondrial fusion protein) and increasing levels of nuclear factor erythroid 2-related factor 2 (a neuroprotective antioxidant) post I/R (85). Thus, dietary levels of curcumin may alleviate retinal I/R injury by restoring mitochondrial function and upregulating the antioxidant system in vulnerable RGCs.

Curcumin has also been found to protect retinal pigment epithelial cells in various cellular models of AMD. In a pulsed H_2_O_2_ model of AMD, curcumin reduced free radicals as well as gene expression of SOD, glutathione, and maleic dialdehyde, in addition to suppressing apoptosis (86). In a light-induced retinal degeneration model of AMD, curcumin was found to inhibit nuclear factor jB and downregulate cellular inflammatory genes (87).

In the diabetic eye, curcumin has been studied as a potential therapeutic for diabetic keratopathy and DR. Guo et al. demonstrated that intranasal nanomicelle curcumin decreased ROS, increased free radical scavengers, increased neurotrophic factors, and decreased mRNA expression of inflammatory cytokines in the corneas of diabetic mice with cornea abrasions (88). In models of DR, curcumin was found to prevent the degeneration of cellular organelles and increase the thickness of retinal capillary basement membranes in diabetic rats (89). Furthermore, curcumin has been found to normalize diabetic microvasculature, decrease tortuosity, shrinkage, narrowing, and microaneurysms with stimulation of regenerative processes in choroidal microvasculature after treatment (90).

Curcumin has been shown in numerous human and animal studies to have an excellent safety profile, with clinical trials reporting no significant adverse effects or organ toxicity for up to 90 days (91, 92) In rat studies lasting 90 days at relatively high oral doses of 500 mg/kg/day, no organ toxicity was observed (93). However, rare instances of curcumin-induced liver toxicity have been highlighted in a few case reports. A systematic review of curcumin-induced liver toxicity noted that adverse events were more likely to occur in high doses of highly bioavailable formulations of curcumin, indicating the need for closer regulation of the production of this supplement (94). Through its potent antioxidant and anti-inflammatory actions, modulation of mitochondrial function and redox signaling, and protective effects on retinal neurons and microvasculature, curcumin may have therapeutic potential to counteract oxidative stress, inflammatory dysregulation, and vascular alterations implicated in the development of SANS, though no spaceflight or spaceflight analogue studies exist. However, clinicians and astronauts should remain aware of the potential for hepatotoxicity in chronic or high-dose use.

### Ubiquinone/Coenzyme Q10

Ubiquinone, also known as Coenzyme Q10 (CoQ10), is a lipid-soluble essential molecule that naturally occurs in every cell membrane in our bodies. It is a common part of the human diet, and it is endogenously synthesized. It plays an essential role in the electron transport chain by shuttling electrons from complexes I & II to complex III, but more importantly CoQ10 is a powerful antioxidant. CoQ10 has been shown to induce expression of SOD and inhibit lipid peroxidation via scavenging of free radicals (95, 96). It can also improve blood flow by preserving NO.

CoQ10 has been studied as a potential therapeutic in AMD and glaucoma (97). A study by Blasi et al. found that patients with AMD (n = 19) had lower plasma levels of CoQ10 compared to age-matched controls (n = 19), suggesting a possible link between AMD and CoQ10 deficiency (98). In another study with 106 patients, 30 mg/day CoQ10 was used as part of a regimen including n-3 fatty acids (1,320 mg/day) and acetyl-L-carnitine (500 mg/day) to treat AMD. They found that the treatment group had significant improvement in visual field mean defect (VFMD), visual acuity, and foveal sensitivity compared to the placebo group after 12 months of treatment (99). Unfortunately, there have been no studies to our knowledge that utilized only CoQ10 as treatment for AMD.

CoQ10 has been used both in rat and human studies as a possible therapeutic for glaucoma. In high IOP rat models, CoQ10 was also shown to prevent RGC loss (100). In glaucomatous mice, CoQ10 supplemented mice increased RGC survival, preserved axons in the optic nerve head, and decreased astroglial activation in the retina and optic nerve head compared to age-matched control mice (101).

CoQ10 has been sold as a dietary supplement in many countries for over 20 years. Numerous studies during this time have examined its safety profile, concluding that CoQ10 is safe in doses of up to 1,200 mg/day/person (102). In rare cases where adverse effects were observed, such as soft stool, constipation, dizziness, and headache, they were deemed clinically insignificant (102). The optimal dose in inflammatory diseases is a daily intake of 300–400 mg, well below the hazardous level of >1,200 mg/day/person. (103). With CoQ10’s high safety profile and through its central role in mitochondrial bioenergetics, potent antioxidant activity, preservation of NO–mediated vascular function and demonstrated neuroprotective effects on RGCs, CoQ10 should be investigated in spaceflight analogues for its potential to mitigate the development of SANS.

### Resveratrol

Resveratrol, or 3,4’,5 trihydroxy-trans-stilbene, is a natural polyphenol found in grape skin. It has been shown to have many beneficial effects, such as the prevention of cardiovascular diseases, anti-cancer potential, neuroprotective effects, maintenance of homeostasis, delaying aging, and decreasing inflammation (104). It works through a variety of mechanisms that give resveratrol its antioxidant, anti-angiogenic, and anti-inflammatory properties. Resveratrol exhibits strong antioxidant activity via upregulation of proteins in the NRF2 and HO-1 pathway (105). In ovariectomized mouse models, it has been demonstrated that resveratrol activates the SIRT1/AMPK antioxidant defense pathways, providing cardiovascular protection (106). The inhibition of angiogenesis by resveratrol is related to its ability to inhibit both vascular endothelial growth factor (VEGF) and hypoxia-inducible factor - 1 generation (107). In addition, resveratrol has been used to suppress leukemia cell proliferation and promote apoptosis of malignant cells by increasing expression of PTEN and inhibiting the PI3K/AKT signaling pathway, which activates apoptosis and reduces angiogenesis (108). However, most of the therapeutic benefit of resveratrol lies in its anti-inflammatory properties.

Resveratrol inhibits inflammation through multiple interconnected signaling pathways that reduce pro-inflammatory gene expression and mediator production. It decreases prostaglandins and thromboxanes via the decreased activity and transcription of Cyclooxygenase-1 (COX-1) and Cyclooxygenase-2 (COX-2), although studies are inconsistent to the degree and selectivity by which resveratrol may inhibit each pathway (109–111). Resveratrol also powerfully blocks the activation of NF-κB via multiple molecular mechanisms, reducing the production of TNF-α, IL-1β, IL-6, and inducible nitric oxide synthase (111).

Resveratrol has shown potential in treating AMD, DR, retinopathy of prematurity (ROP), and uveitis (112). Within *in vitro* models of AMD using human retinal pigment epithelial cells, resveratrol has shown to inhibit VEGF expression (113). Additionally, a 24-month observation study found that wet-AMD patients receiving resveratrol required fewer anti-VEGF injections and had a lower frequency of fibrosis progression relative to controls (114). In diabetic retina models of DR, resveratrol was shown to decrease oxidative stress markers like Ox-LDL and block apoptosis via the inhibition of caspase-3 (115). Unfortunately, there is a lack of human studies verifying the efficacy of resveratrol as a treatment for DR in a clinical setting.

In a study of endotoxin induced uveitis in rats, five days of resveratrol prophylaxis had shown to significantly decrease the inflammatory markers Intercellular Adhesion Molecule-1 (ICAM-1) and Monocyte chemoattractant protein-1 (MCP-1) (116). In several rat studies on ROP, resveratrol has been shown to decrease markers of retinal injury, as well as inhibit angiogenesis and apoptosis (117–119). Once again, there is unfortunately a lack of human studies that examine the use of resveratrol for the treatment of uveitis and ROP in humans.

In studies that examined the safety profile of resveratrol, resveratrol has been shown to be non-toxic in doses as high as 700 mg/kg/day in rats (120). The study by Williams et al. found no adverse effects in the skin/eyes, genome, reproductive tracts, or liver after 28 days (120). In a meta-analysis that examined resveratrol as an adjunct for diabetes mellitus type II treatment, resveratrol therapy was associated with a statistically significant improvement in cardiac biomarkers and no increase in adverse effects relative to placebo groups was observed (121). Due to its high safety profile and its potent antioxidant and anti-inflammatory effects, modulation of vascular and angiogenic signaling, and demonstrated neuroprotective actions in retinal tissues, resveratrol should be investigated in SANS models as a possible preventative therapy for SANS.

### Vitamin E

Vitamin E (tocopherol) is dietary antioxidant abundantly found in green leafy vegetables, nuts, seeds, and plant oils (122). Tocotrienols are a precursor to tocopherols that penetrate the lipid bilayer of cells much more rapidly, increasing the effectiveness of Vitamin E as an antioxidant (122). Vitamin E has been shown to exhibit anti-radiation and anti-inflammatory properties in addition to acting as an antioxidant. α-Tocopherol has been shown to prevent radiation-induced cytopenia by halting lipid peroxidation reactions, while δ-tocotrienol has been shown to shield mouse bone marrow and human CD34+ cells from radiation damage, via the activation of Erk (123–125). Furthermore, various forms of Vitamin E have exhibited anti-inflammatory effects through a variety of mechanisms. In one study, α-tocopherol lowered the inflammatory level as observed by reduced macrophage infiltration and JNK/c-Jun signaling while another study demonstrated that γ-tocopherol efficiently traps inflammation-induced electrophiles (126, 127). Through these properties, Vitamin E has been shown to treat various eye diseases.

Across a range of experimental and review studies, both tocopherols and tocotrienols have been investigated for their potential role in ocular pathologies driven by oxidative stress and neurodegeneration. When investigating their role in glaucoma, a narrative review that integrated omics data concluded that tocopherols and tocotrienols may modulate oxidative stress, mitochondrial dysfunction, and inflammatory signaling related to this disease (122). When the same author conducted a comprehensive review that summarized preclinical evidence, they found that Vitamin E isoforms may protect RGCs, reduce oxidative damage, and modulate inflammatory and apoptotic pathways necessary to glaucoma pathogenesis (128). In both studies, the authors note a lack of robust clinical evidence in treatment of glaucoma. In galactosemic rats, topically applied tocotrienol at concentrations 0.01%-0.05% was shown to delay cataract formation and improve lens redox balance by reducing lipid peroxidation and restoring antioxidant capacity, supporting a protective role in cataractogenesis; however, 0.20% concentration of applied tocotrienol aggravated cataractogenesis (129). Duncan et al. conducted an *in vitro* experimental study using human retinal pigment epithelium cells, demonstrating that tocotrienols and certain tocopherols provide significant protection through antioxidant and membrane-stabilizing mechanisms in a model of AMD (130). Unfortunately, there is a lack of human clinical evidence to support the routine addition of vitamin E to the therapeutic regimens of these pathologies.

Vitamin E has a generally favorable safety profile when used at dietary or modest supplemental doses. According to the NIH Office of Dietary Supplements, the upper tolerable intake level for adults is 1,000 mg/day, while most supplements are 100–400 mg/day. The primary concern with Vitamin E overdose is hemorrhagic complications from Vitamin E antagonizing Vitamin K-dependent clotting factors and inhibiting platelet aggregation (131). While this is a concern for long-term overdose of Vitamin E, careful dosing and monitoring can easily prevent this complication. Overall, Vitamin E’s therapeutic properties may have therapeutic potential to mitigate radiation-induced oxidative damage, inflammatory dysregulation, and neuro-ocular injury implicated in the development of SANS and should be investigated in spaceflight models.

### B vitamins

B-complex vitamins are a group of water-soluble vitamins that are excreted in the urine and need to be replenished daily. They play essential roles in catabolic and anabolic metabolism, and deficiencies in these vitamins can lead to severe adverse effects. Mutations in one-carbon metabolism pathways, in which B-complex vitamins play an important role in, have been identified as potentially predisposing astronauts to the earlier and more severe development of SANS (12). While the exact mechanism behind the contribution of one-carbon metabolism genetic variants and B-vitamin status to SANS development has yet to be fully elucidated, alterations in both has been hypothesized to lead to endothelial dysfunction and increased vascular permeability, which has been found to worsen under conditions of weightlessness (12). This makes B-Vitamins particularly interesting as possible preventative agents for SANS. While there are eight different compounds in the B-vitamin family, only thiamine (B_1_), pyridoxine (B_6_), folic acid (B_9_), and cobalamin (B_12_) have shown promise in treating ocular pathologies as opposed to vitamins B_2_, B_3_, B_5_, and B_7_, which are primarily used clinically to correct their respective deficiencies.

Thiamine (B_1_) is absorbed in the duodenum and gets converted to its active form thiamine pyrophosphate (TPP). TPP is a cofactor for crucial enzymes in the citric acid cycle, hexose monophosphate (HMP) shunt, and amino acid breakdown pathways. Low thiamine levels have been shown to cause altered mitochondrial activity, impaired oxidative metabolism, and decreased energy production (132). Neurons are particularly predisposed to damage from thiamine deficiency due to their high energy demand, and chronic thiamine deficiency can lead to irreversible memory loss in the form of Wernicke-Korsakoff syndrome (133). TPP plays an essential role in the production of acetylcholine and myelin, as well as the maintenance of glutamate, aspartate, and gamma-aminobutyric acid levels (132). The evidence for thiamine’s role in preventing ocular diseases is limited to just one cross-sectional comparative study that found that diabetic patients with lower levels of thiamine had a higher risk of DR (134, 135).

Pyridoxine (B_6_) is found in beef, poultry, starchy vegetables, and non-citrus fruits, and is converted to its active form pyridoxal 5’ phosphate (136). Pyridoxal 5’ phosphate plays essential roles in heme synthesis, maintenance of normal homocysteine levels, supporting immune function and brain health, and the breakdown of proteins, carbohydrates, and lipids (133). Deficiencies in pyridoxine can lead to microcytic anemia, dermatological abnormalities, electroencephalogram abnormalities, depression, confusion, weakened immune function, and glossitis (133). The role of pyridoxine in multiple ocular pathologies has been investigated, but the studies are not as robust and often do not isolate pyridoxine itself. In a longitudinal double-masked, randomized, placebo-controlled trial it was found that women at high risk of cardiovascular disease had a lower chance of developing AMD when supplemented with daily supplementation of B_6_/B_9_/B_12_ compared to placebo (137). When pyridoxine was investigated as possible supplemental therapies for glaucoma and cataracts, studies showed no improvement in ocular health when patients were supplemented with daily pyridoxine (138, 139). While the role of pyridoxine is controversial, when used in combination with other B-vitamins it could serve a valuable role as a preventative agent in SANS.

Folic acid (B_9_) is found primarily in leafy green vegetables and plays major roles in nucleic acid synthesis, erythropoiesis, and homocysteine metabolism (133). Deficiencies in folic acid have been associated with megaloblastic anemia, oral ulcerations, and dermatological changes, as well as congenital birth defects in children born to deficient mothers (133). In addition to its potential role of lowering the chance of developing AMD in susceptible patients, higher total intake of folic acid was associated with a lower risk for developing exfoliative glaucoma in a prospective cohort study of 399 patients over the span of 20 years (139).

Cobalamin (B_12_) is found in animal products and is absorbed in the distal ileum, where it combines with intrinsic factor prior to absorption (133). Similar to folic acid, it is required for erythropoiesis, but it also plays roles in neurologic function, myelination of neurons, and the synthesis of DNA and RNA (133). Thus, cobalamin deficiency can lead to megaloblastic anemia, fatigue, low appetite, and neuropsychiatric complications ranging from paresthesias to subacute combined degeneration (133). The efficacy of cobalamin supplementation for the prevention of ocular pathologies has shown mixed results, with the supplementation of cobalamin showing no decreased risk of developing exfoliative glaucoma or cataracts, whereas cobalamin in a regimen including B_6_ and B_9_ appeared to decrease risk of developing AMD and DR later in life (135, 137–139).

Despite their mixed results in terrestrial ocular pathologies, folic acid and cobalamin are of particular interest in SANS due to the emerging hypothesis that certain variants in the folate- and cobalamin-dependent one-carbon pathways may predispose astronauts to developing many of the neuro-ocular signs seen in SANS (140). In this study, Zwart et al. found that serum homocysteine, cystathionine, 2-methylcitric acid, and methylmalonic acid concentrations were increased 25-45% higher in astronauts with clinically detectable SANS findings compared to those without them after LDSF (140). Notably, these differences were observed before, during, and after spaceflight, which further points toward a possible genetic predisposition to developing SANS (140). In a later study, Zwart et al. identified associations between methionine synthase reductase (MTRR) and serine hydroxymethyltransferase (SHMT1) polymorphisms and spaceflight-induced vision changes (141). Specifically, they found that the minor MTRR 66G allele was associated with the development of CF and cotton-wool spots (141). Additionally, the SHMT1 1420C allele increased likelihood of developing clinically significant ODE, while the SHMT1 1420T allele appeared to be protective against ODE (141). Furthermore, B-vitamin status significantly predicted ocular outcomes, indicating that B-vitamin supplementation may play a protective role in genetically predisposed astronauts (141).

B-vitamin supplementation is generally known to have a high safety margin and wide therapeutic index, primarily due to their water-solubility and ability to excrete excess amounts in urine. According to the NIH Office of Dietary Supplements, toxicity for these vitamins is rare at doses common in clinical practice (133). Notable exceptions include: vitamin B_6_, which has been associated with sensory neuropathy in chronic, high doses; vitamin B_3_, which can cause flushing, hepatotoxicity, hyperglycemia, and hyperuricemia, even at pharmacologic doses; and vitamin B_9_, which when taken in excess can mask vitamin B_12_ deficiency (133). However, B-vitamin supplementation is considered safe and well-tolerated for most patients at doses within established tolerable upper intake levels.

Despite the theoretical rationale for B-vitamin supplementation for SANS prevention, NASA has not recommended this routinely. Current NASA nutritional standards for exploration missions define specific dietary requirements and acceptable intake ranges rather than endorsing open-ended supplementation (142). Specifically, there has been only one documented attempt at in-flight B-vitamin supplementation for SANS reported on an astronaut with two predisposing one-carbon metabolism risk factors who received 100 mg B6, 5 mg methylfolate, 1000μg B12, and 400 mg riboflavin beginning on flight day 153 through 169, when she stopped due to GI discomfort (143). This case showed subsequent improvement in SANS findings, but distinguishing between natural disease progression, B-vitamin supplementation, and reduction in cabin CO_2_ would require future studies (143). Additionally, SANS findings had already been detected at flight day 30 before supplementation began, and total retinal thickness and surface roughness began decreasing prior to B-vitamin supplementation (143). This case underscores both the tolerability challenges and likely multifactorial approach that could be necessary for the prevention and treatment of SANS.

### Summary of compounds

Taken together, the compounds reviewed here should not be viewed as eight independent candidates but as agents converging on a small, interconnected set of mechanistic targets relevant to SANS. Their common denominator is the mitigation of oxidative stress and its downstream consequences, but they distribute across four overlapping domains. The first is direct antioxidant and mitochondrial protection, which is most prominent for CoQ10, Vitamin E, curcumin, saffron/crocin, and resveratrol, which all counter ROS generated by GCR and microgravity-induced mitochondrial dysfunction. The second is endothelial and microvascular regulation, led by Mirtogenol and GBE with additional contributions from saffron and resveratrol, targeting the impaired ocular perfusion and venous congestion described in SANS through enhanced NO signaling and improved blood flow. Third, they suppress inflammation, primarily via NF-κB inhibition through curcumin, resveratrol, saffron/crocin, and GBE. Finally, B-vitamins support one-carbon metabolism, which has been genetically and biochemically implicated in SANS (140, 141). Because these domains are interconnected, compounds acting across several domains in combination are more likely to succeed than any single agent targeting oxidative stress in isolation.

## Practical considerations for supplement-based countermeasures

While the preceding sections establish a biological rationale to examine the use of naturally occurring antioxidant and anti-inflammatory compounds in the prevention of SANS, it is important to acknowledge the practical challenges that must be addressed in LDSF.

### Compound stability and shelf life

A critical barrier to any pharmacological countermeasure for LDSF is long-term stability. Currently, medications aboard the ISS are replaced before expiration through routine resupply; however, resupply will not be possible during missions to Mars or beyond and therefore it is desired that any intervention have a shelf life that lasts the duration of the mission (144). Comparisons between the shelf life of terrestrial controls and pharmaceuticals flown in space missions demonstrate that there is decreased efficacy of medications that worsens as a function of time in space (145). This may be due to unique environmental factors to space including microgravity, CO_2_ levels, excessive vibration, hard vacuum, humidity variation, temperature differences, and excessive radiation (144, 146). Furthermore, the altered degradation of pharmaceuticals may expose astronauts to hazardous impurities that could negatively impact their health (146). Any proposed countermeasure must therefore demonstrate adequate stability over the full mission duration, and innovative strategies in packaging, and storage should be implemented to prevent degradation (144).

### Payload logistics and resource constraints

LDSF missions face strict mass, volume, and power limitations for all onboard resources (147). Quantitative trade space analysis tools developed by NASA, such as the Medical Optimization Network for Space Telemedicine Resources (MONSTR), have been designed to prioritize resource inclusion by identifying the most flexible and intersecting elements that maximize medical capability available to exploration crews while minimizing mass, volume, and crew burden (147). This framework favors resources with utility across multiple conditions, for example, anti-inflammatory agents were identified as relevant to 22 distinct medical conditions modeled for a 2.5 year Mars transit mission (147). Many of the agents that were discussed in this article could prove to have beneficial effects outside of SANS, which could reduce the total mass and volume of the payload required for each mission. For a summary of compound dosages in human studies as a possible reference for what may be used in LDSF, see **Table 3**.

**Table 3:** Summary of compound dosages in humans.

Compound	Effective dose in cited human study	Safety ceiling/upper intake	References
Mirtogenol^®^	80 mg standardized bilberry extract + 40 mg Pycnogenol daily	No side effects observed over 6 mo	(44)
Ginkgo biloba extract	160 mg/day (80 mg BID); 120 mg/day (40 mg TID)	No significant AEs vs placebo across 80–600 mg/day; coagulation unchanged at 240 mg/day	(55, 56, 66)
Saffron (whole extract)	20 mg/day	Doses <1.5 g/day without excess AEs vs placebo	(67, 73)
Crocin (saffron constituent)	15 mg/day	Well tolerated at 15 mg/day over 3 mo	(71)
Curcumin	No human ocular-efficacy dose cited (preclinical/cell models only)	No organ toxicity in 90-day human/rat safety studies; rare hepatotoxicity with high-dose bioavailable formulations	(91, 93, 94)
Coenzyme Q10	30 mg/day (in combination with acetyl-L-carnitine + n-3 FAs)	Safe up to 1200 mg/day; optimal anti-inflammatory 300–400 mg/day	(99, 102, 103)
Resveratrol	60 mg/day	Nontoxic to 700 mg/kg/day in rats; no excess AEs in T2DM meta-analysis	(114, 120, 121)
Vitamin E	No human ocular-efficacy dose cited (preclinical/cell models only)	Tolerable UL 1000 mg/day (NIH ODS); bleeding risk from vitamin K antagonism at high doses	(131)
B vitamins (B6/B9/B12)	Astronaut case: B6 100 mg, methylfolate 5 mg, B12 1000 µg, riboflavin 400 mg	Water-soluble, wide margin; B6 sensory neuropathy at chronic dose as low as 50 mg/day	(137, 143)

Summary of the naturally occurring compounds reviewed as candidate SANS countermeasures, listing the effective dose reported in the cited human study, the corresponding safety ceiling or tolerable upper intake level, and supporting references. Effective doses are those associated with ocular or clinical benefit in the cited human studies; for curcumin and vitamin E, no human ocular-efficacy dose is listed because supporting evidence derives from preclinical or cell-based models only, and for coenzyme Q10 and the B vitamins the effective dose was administered as part of a multi-component regimen rather than as monotherapy. Safety ceilings reflect the highest doses shown to be well tolerated, established tolerable upper intake levels, or dose-related adverse effects reported in the cited sources; these derive from heterogeneous populations and study designs and are not spaceflight-specific. Doses and safety thresholds should therefore be interpreted as terrestrial reference points requiring validation in spaceflight or analogue environments. Abbreviations: SANS, spaceflight-associated neuro-ocular syndrome; AE, adverse event; BID, twice daily; TID, three times daily; UL, tolerable upper intake level; NIH ODS, National Institutes of Health Office of Dietary Supplements; T2DM, type 2 diabetes mellitus; n-3 FAs, omega-3 fatty acids.

### Altered pharmacokinetics in spaceflight

Spaceflight induces physiological changes that may alter the absorption, distribution, metabolism, and excretion of orally administered compounds. These changes include fluid shifts, changes in GI motility, and alterations in hepatic metabolism that may affect drug and supplement bioavailability and clearance (148–150). Most medications used during spaceflight are administered under the assumption that they act similarly to the way they do on earth, as pharmacokinetic studies during spaceflight are limited (149). This knowledge gap also applies to natural supplements, and pharmacokinetic studies in spaceflight or high-fidelity analogue environments should be performed prior to establishing any supplement-based countermeasure.

## Conclusion

SANS represents a complex, multifactorial challenge that threatens the feasibility and safety of human space exploration. Despite over a decade of investigation, no single unifying mechanism or universally effective countermeasure has been identified. While current treatment strategies primarily focus on mechanical or physiologic approaches to mitigating elevated ICP and venous congestion, our growing understanding of SANS reveals that metabolic, oxidative, inflammatory, and microvascular pathways play important contributory roles to SANS pathophysiology. These pathways have also been implicated in certain terrestrial ocular diseases, providing a strong rationale for exploring compounds with established ocular effects as potential adjuncts to preventative regimens. Given the unique difficulty of finding therapeutics suitable to the dangerous environment of space, naturally occurring compounds with high therapeutic indices show great potential in serving adjunctive roles.

In this review, we examined a range of natural compounds that have demonstrated antioxidant, anti-inflammatory, neuroprotective, and vasoregulatory effects in preclinical models and certain human ocular disease studies. Collectively, these agents target mechanisms highly relevant to SANS, such as oxidative stress from GCR, endothelial dysfunction, impaired venous and lymphatic outflow, mitochondrial injury, altered angiogenesis, and one-carbon metabolic disturbances (**Figure 1**). Nevertheless, translation of these findings to SANS remains speculative since direct studies in spaceflight or analog environments are notably lacking. Future research should prioritize well-designed ground-based analog studies and controlled spaceflight investigations when feasible. For a complete summary of the quality of evidence for each of these compounds, please reference **Table 4**.

**Figure 1:**
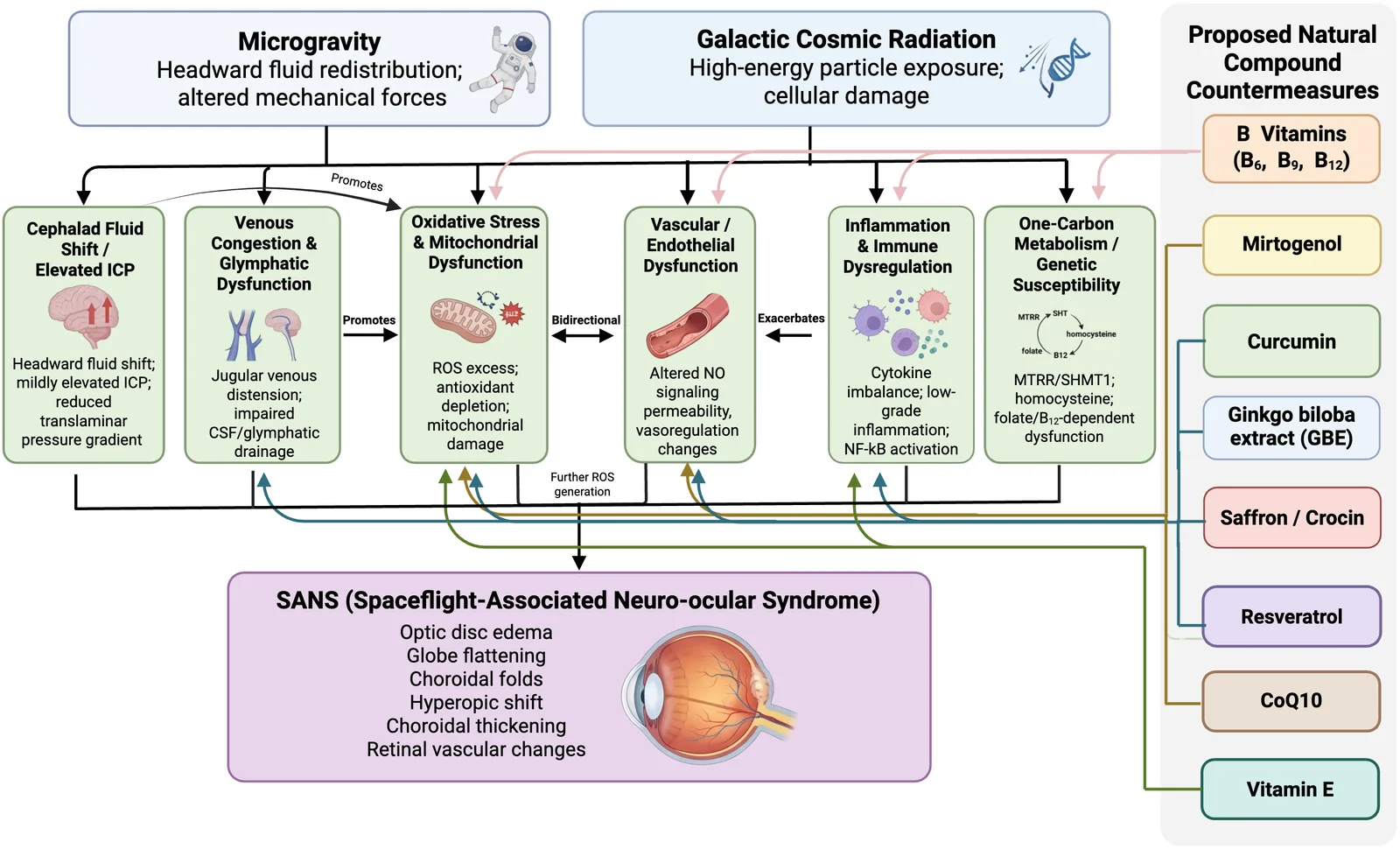
Pathophysiological mechanisms linking spaceflight to spaceflight-associated neuro-ocular syndrome and potential targets of natural compounds. Schematic illustrating the proposed pathophysiological pathways through which spaceflight contributes to the development of SANS, including alterations in intracranial and intraocular pressure, oxidative stress, vascular dysfunction, inflammation, mitochondrial dysfunction, and disrupted one-carbon metabolism. The figure also maps the natural compounds reviewed in this manuscript onto the specific pathways they are proposed to modulate, highlighting antioxidants that target oxidative stress, compounds that improve vascular function, and agents that support one-carbon metabolism and related cellular processes.

**Table 4:** Summarization of data and quality.

Compound	Controlled trials (human)	Uncontrolled/observational human studies/reviews	Preclinical *in vivo* (animal)	*In vitro*/cell models	Spaceflight or analogue studies	References
Mirtogenol (Bilberry + Pine Bark)	Prospective controlled trial: reduced IOP from 25.2 to 22.0 mmHg over 2–3 months with improved ocular blood flow without adverse effects; no increased adverse events vs. placebo	Pine bark (Pycnogenol): reduced retinal edema and improved vascular flow in early DR; bilberry extract	Wistar glaucoma model: reduced IOP and significantly decreased RGC apoptosis	None	None	(42, 44, 45, 49)
Ginkgo Biloba Extract (GBE)	Prospective placebo-controlled crossover RCT (n=28, 4 weeks): no significant VF improvement in NTG; controlled studies: increased ophthalmic artery end-diastolic velocity and retrobulbar blood flow vs. placebo; RCT (n=50): no changes in coagulation or platelet aggregation	Long-term observational (4 years): significant VF improvement in NTG with no IOP change; systematic reviews: no significant difference in adverse events between placebo and 80–600 mg GBE; systematic review: possible association with increased bleeding risk	Rat chronic glaucoma model: decreased RGC loss; rodent studies: increased choroidal blood flow	Neuroprotection of cultured human RGCs	None	(50, 55 –61, 65, 66)
Saffron/Crocin	RCT in mild/moderate AMD: preserved visual acuity; double-blind placebo-controlled RCT in AMD: improved retinal flicker sensitivity; pilot RCT in POAG: significantly decreased IOP after 3 weeks as adjunct to timolol/dorzolamide; placebo-controlled RCT in diabetic maculopathy: improved CMT, BCVA, hyperglycemia, and HbA1c at 3 months	Safety data: doses <1.5 g/day not associated with increased adverse events vs. placebo	Rat and rabbit studies: crocin/crocetin enhanced oxygen diffusion and improved ocular blood flow	None	None	(67, 70, 71, 73–76)
Curcumin	Safety trials: no significant adverse effects or organ toxicity for up to 90 days in human clinical trials	Systematic review: rare hepatotoxicity cases, more likely with high-dose highly bioavailable formulations	Rat high-IOP model: increased BV-2 microglia viability; rat glaucoma I/R model: neuroprotective via restored mitochondrial function and Nrf2 upregulation; diabetic mice: intranasal nanomicelle curcumin decreased ROS and inflammatory cytokines in corneas; diabetic rats: prevented organelle degeneration, increased retinal capillary basement membrane thickness, normalized choroidal microvasculature; rat 90-day safety: no organ toxicity at 500 mg/kg/day	H2O2-pulsed RPE cells (AMD model): reduced free radicals and suppressed apoptosis; light-induced retinal degeneration model: inhibited NF-kB and downregulated inflammatory genes	None	(84–94)
Coenzyme Q10 (CoQ10)	RCT in AMD (n=106): CoQ10 (30 mg/day) + n-3 fatty acids + acetyl-L-carnitine improved VFMD, visual acuity, and foveal sensitivity vs. placebo at 12 months (combination regimen; CoQ10 not isolated)	Case-control (n=38): AMD patients had lower plasma CoQ10 vs. age-matched controls; safety data: safe up to 1,200 mg/day with only clinically insignificant adverse effects	High-IOP rat model: prevented RGC loss; glaucomatous mice: increased RGC survival, preserved optic nerve head axons, decreased astroglial activation	None	None	(98–103)
Resveratrol	None	24-month observational study in wet AMD: fewer anti-VEGF injections and reduced fibrosis progression vs. controls; Meta-analysis of RCTs in type 2 diabetes: improved cardiac biomarkers with no increase in adverse effects vs. placebo (no ocular-specific controlled trials)	Rat endotoxin-induced uveitis: decreased ICAM-1 and MCP-1 after 5-day prophylaxis; multiple rat ROP models: decreased retinal injury markers, inhibited angiogenesis and apoptosis; diabetic retina models: decreased Ox-LDL and blocked caspase-3-mediated apoptosis; safety: non-toxic at 700 mg/kg/day in rats for 28 days	Human RPE cells (AMD model): inhibited VEGF expression	None	(113–121)
Vitamin E	None for ocular indications	None for ocular indications	Galactosemic rats: topical tocotrienol (0.01–0.05%) delayed cataract formation and improved lens redox balance; however, 0.20% concentration aggravated cataractogenesis; mouse bone marrow: tocotrienol shielded from radiation damage via Erk activation; tocopherol: prevented radiation-induced cytopenia; tocopherol: reduced macrophage infiltration and JNK/c-Jun signaling in inflammatory model	Human RPE cells (AMD model): tocotrienols and certain tocopherols provided antioxidant and membrane-stabilizing cytoprotection; human CD34+ cells: tocotrienol protected from radiation damage	None	(122–131)
B Vitamins (B1, B6, B9, B12)	RCT (B6/B9/B12 combination): women at high CV risk had lower AMD incidence vs. placebo; RCT: B6/B9/B12 showed no benefit for cataracts; prospective cohort (n=399, 20 years): higher folic acid intake associated with lower exfoliative glaucoma risk; RCT: B6 alone showed no benefit for glaucoma	Cross-sectional: diabetic patients with lower thiamine levels had higher DR risk; astronaut biomarker study: SANS-affected astronauts had 25–45% higher homocysteine, cystathionine, 2-methylcitric acid, and methylmalonic acid vs. unaffected astronauts; genetic association study: MTRR 66G and SHMT1 1420C polymorphisms associated with SANS findings; B-vitamin status predicted ocular outcomes	None	None	Single case report (in-flight): astronaut with 2 one-carbon metabolism risk factors received B6 (100 mg), methylfolate (5 mg), B12 (1000?g), riboflavin (400 mg) from flight day 153–169; subsequent improvement in SANS findings, but confounded by natural disease progression and reduced cabin CO; discontinued due to GI discomfort	(134, 135, 137–143)

Summary of the evidence base supporting each reviewed compound as a candidate SANS countermeasure, stratified by level of evidence: controlled human trials, uncontrolled or observational human studies and reviews, preclinical in vivo (animal) studies, in vitro/cell-based models, and spaceflight or analogue studies. This tiered organization is intended to make explicit the strength and directness of the evidence for each compound and to highlight where support derives from terrestrial ocular disease rather than SANS itself. Notably, the vast majority of human evidence comes from studies of glaucoma, age-related macular degeneration, and diabetic retinopathy rather than SANS; several entries (e.g., coenzyme Q10, B vitamins) reflect multi-component regimens in which the individual compound was not isolated. Findings should therefore be interpreted as indirect, hypothesis-generating support requiring validation in spaceflight-relevant models. Abbreviations: SANS, spaceflight-associated neuro-ocular syndrome; IOP, intraocular pressure; RGC, retinal ganglion cell; DR, diabetic retinopathy; AMD, age-related macular degeneration; NTG, normal-tension glaucoma; POAG, primary open-angle glaucoma; VF, visual field; CMT, central macular thickness; BCVA, best-corrected visual acuity; HbA1c, glycated hemoglobin; VFMD, visual field mean defect; RPE, retinal pigment epithelium; I/R, ischemia/reperfusion; ROP, retinopathy of prematurity; ROS, reactive oxygen species; VEGF, vascular endothelial growth factor; ICAM-1, intercellular adhesion molecule-1; MCP-1, monocyte chemoattractant protein-1; Ox-LDL, oxidized low-density lipoprotein; MTRR, methionine synthase reductase; SHMT1, serine hydroxymethyltransferase 1; CV, cardiovascular; n-3, omega-3; GI, gastrointestinal; RCT, randomized controlled trial.

This review produces two specific, testable hypotheses. First, that pre- and in-flight supplementation with an antioxidant/vasoregulatory compound reduces the incidence or severity of ODE and choroidal thickening relative to placebo. Second, that combining a metabolically targeted compound with a mechanical countermeasure such as lower body negative pressure produces additive protection against choroidal engorgement in SANS (39).

These hypotheses can be tested across multiple models. Contrary to early assumptions, SANS-relevant ocular changes can be at least partially modeled in animals. For example, a handlimb-unloading mouse model of headward fluid shift reproduces transient IOP elevation and retinal nerve fiber layer thinning, providing a tractable platform for dosing, pharmacokinetic, and mechanistic studies of candidate compounds (151). Rodent models also allow controlled exposure to simulated GCR, enabling evaluation of radioprotective and antioxidant compounds against the oxidative component of SANS that cannot be isolated in human analogues (13). In humans, strict 6° head-down tilt bed rest reproduces ODE and posterior globe flattening and is the best-validated terrestrial analogue for testing countermeasure efficacy, although it does not fully replicate the choroidal thickening seen in LDSF and may exaggerate ICP relative to microgravity (152–154). Strict supine (0°) bed rest more closely approximates the mild ICP elevation of microgravity and has been used successfully to test countermeasures against acute choroidal engorgement (39).

These studies should evaluate compounds individually and in combination, focusing on dosing, pharmacokinetics in microgravity, genetic predispositions, and clinical SANS findings. Given the multifactorial nature of SANS pathophysiology, a multimodal preventative strategy that incorporates safe, metabolically targeted compounds in combination with mechanical interventions is likely to prove most effective. Advancing this line of investigation has the potential not only to mitigate SANS risk but also to deepen our understanding of neuro-ocular physiology under extreme conditions and improve ocular health strategies both in space and on Earth.
